# The impact of tracheotomy on levels of procalcitonin in patients without sepsis: a prospective study

**DOI:** 10.6061/clinics/2015(09)03

**Published:** 2015-09

**Authors:** Xingui Dai, Chunlai Fu, Changfa Wang, Yeping Cai, Sheng'an Zhang, Wei Guo, Daibing Kuang

**Affiliations:** I The First People's Hospital of Chenzhou, Institute of Translational Medicine, Department of Critical Care Medicine, Chenzhou, Hunan, China.; II Central South University, Third Xiangya Hospital, Department of General Surgery, Changsha, Hunan, China.

**Keywords:** Tracheotomy, Procalcitonin, Sepsis, Percutaneous Dilatational Tracheotomy, Surgical Tracheotomy

## Abstract

**OBJECTIVE:**

Procalcitonin is a reliable biomarker of infection and sepsis. We aimed to determine whether tracheotomy influences the procalcitonin concentrations in patients without sepsis and assess whether operative duration and procedure affect the peak procalcitonin level.

**METHODS:**

A total of 38 non-septic patients who required a tracheotomy underwent either a percutaneous dilatational tracheotomy (n=19) or a surgical tracheotomy (n=19). Procalcitonin levels were measured at the beginning of the tracheotomy and at 2 h, 4 h, 8 h, 24 h, 48 h and 72 h after the procedure.

**RESULTS:**

The baseline procalcitonin concentration before the tracheotomy was 0.24±0.13 ng/mL. The postoperative levels increased rapidly, with a 4-fold elevation after 2 h, reaching a peak 4 h later with a 5-fold increase over baseline. Thereafter, the levels gradually returned to 2-fold greater than the baseline level within 72 h. The peak levels of procalcitonin showed a significant positive correlation with operative durations (r=0.710, *p*<0.001) and procedures (rho=0.670, *p*<0.001).

**CONCLUSION:**

In patients without sepsis, tracheotomy induces a rapid release of serum procalcitonin, and the operative duration and procedure have significant impacts on the peak procalcitonin levels. Thus, the nonspecific increase in procalcitonin levels following tracheotomy needs to be considered when this measure is used to evaluate infection.

## INTRODUCTION

Procalcitonin (PCT), a soluble protein comprising 116 amino acids with a sequence identical to that of the calcitonin prohormone, has been considered a reliable biomarker for the evaluation of bacterial infection and sepsis 1,2. In healthy individuals, serum PCT concentrations are undetectable 3, but in cases of severe bacterial infection and sepsis, PCT concentrations increase rapidly. PCT is mainly released by peripheral blood mononuclear cells, and the levels of PCT are modulated by bacterial lipopolysaccharides (LPS) and sepsis-associated cytokines 4,5. At present, PCT is widely used for detecting sepsis, evaluating the severity of illness and guiding antibiotic administration in the intensive care unit (ICU). However, PCT is induced in the plasma of patients not only in the presence of a bacterial infection but also under conditions of stress such as severe trauma or surgery. Under these conditions, the increase in PCT is moderate, with a rapid decrease to the baseline level 6-9. Other conditions with elevated PCT concentrations include medullary thyroid carcinoma 10, cancer 11, anaphylactic shock 12, heat shock 13, cardiac arrest 8 and Kawasaki disease 14. These findings suggest that a nonspecific elevation in PCT levels can be challenging in clinical practice. For example, a PCT-guided strategy for antibiotic administration is controversial. Although increasing evidence from a variety of studies has shown a promising reduction in the number of antibiotic exposures, duration of antibiotic therapy and even length of stay in the ICU 15,16, several studies have shown that a PCT-guided protocol may not impact the prescription rate of antibiotics or the outcome of patients with infection 17,18.

Tracheotomy is one of most commonly performed surgical procedures and is used to ensure airway clearance by creating a hole in the front of the trachea in the ICU. However, studies have shown that surgical procedures to the neck area cause an increase in thyroid hormones due to surgical stress 19,20. This process may be due to a variety of clinical manifestations leading to an alteration in hormone levels or even a thyroid storm 21. To the best of our knowledge, no study has addressed the potential impact of tracheotomy on the PCT concentration. Thus, the aim of our study was to evaluate whether tracheotomy affects PCT concentrations in patients without sepsis. Moreover, we assessed whether the operative duration and procedure are correlated with peak PCT levels.

## MATERIALS AND METHODS

### Ethics

This prospective observational study was conducted in the general ICU of the First People's Hospital of Chenzhou, Hunan Province, PR China. The study was approved by the Ethics Committee of our hospital and was registered with the U.S. National Institutes of Health Clinical Trials Register (NCT02406079). Written informed consent was obtained from all participating patients or their family members.

### Study design and participants

All prospective participants were selected from among inpatients admitted to the ICU between March 2013 and March 2014. To evaluate the impact of operative procedures on PCT concentrations, the patients were randomly divided into two groups, a percutaneous dilatational tracheotomy (PDT) group (n=19) and a surgical tracheotomy (ST) group (n=19), according to an allocation sequence generated from a computerized random number table. Antibiotic therapy was performed on postoperative patients who either developed fever (temperature >38.5°C) or had an increase in sputum.

### Inclusion and exclusion criteria

Prospective adult patients (18 years and older) who did not have sepsis and who required a tracheotomy were assessed for inclusion in the study. The following patients were excluded: (1) those who did not give their consent or declined treatment during the period of observation; (2) those with a history of thyroid disease, such as hyperthyroidism, hypothyroidism, or thyroid tumors; (3) those who had received or were receiving high-dose steroid treatment; (4) those who received renal replacement therapy (RRT); (5) those with severe renal injury (Scr > 300 umol/L); and (6) those who received antibiotic therapy from 48 h pre-operation to 72 h post-operation.

### Definitions of sepsis

Based on the diagnostic criteria advanced by the 2001 International Sepsis Definition Conference 22, sepsis is a host response to infection leading to systemic inflammatory response syndrome (SIRS), which is defined as the presence of two or more of the following conditions: hypothermia or fever (body temperature <36°C or >38.5°C, respectively); tachycardia (>90 beats/min); tachypnea (>20 breaths/min or PaCO_2_ <32 mmHg when on mechanical ventilation); and leukocytosis (>12,000/mm^3^), leukopenia (<4,000/mm^3^), or an increased number of immature band forms (>10%).

### Clinical data collection

Upon admission to the ICU, data on the patients' baseline characteristics were collected, including age, gender, etiological factors, and underlying diseases. In addition, other physiological and clinical information was collected and scored using the Acute Physiology and Chronic Health Evaluation (APACHE) II criteria. Duration of the operation for those patients who had undergone a tracheotomy was also recorded. The duration was defined as the time when local anesthetics were first administered (2% lidocaine) to the time when skin closure was completed.

### Measurement of plasma PCT

Blood samples for PCT were obtained at the beginning of the tracheotomy (T0) and 2 h (T2), 4 h (T4), 8 h (T8), 24 h (T24), 48 h (T48) and 72 h (T72) after the operation. Serum concentrations of PCT were measured using an electrochemiluminescence immunoassay (ECLIA) method (Roche Diagnostic, GMBH, Mannheim, Germany) with a detection limit of 0.02 ng/mL; a concentration<0.02 ng/mL was defined as 0.02 ng/mL.

### Statistical analysis

The results for continuous variables with normal distributions were recorded as the mean±standard deviation (SD). The student's *t*-test for unpaired samples was used to compare the mean values of the two groups. The PCT changes over time were compared using a repeated measure analysis of variance (ANOVA). The results for categorical variables were expressed as n (%) and compared using Pearson Chi-square tests or Fisher's exact tests when the expected value was less than 5. The correlation between the peak PCT level and the operation duration was assessed using the Pearson correlation coefficient, whereas the correlation between the peak PCT level and the operative procedure was analyzed using Spearman's rank correlation coefficient. Statistical analyses were conducted using IBM SPSS 19.0 (SPSS, Chicago, IL, USA), and a *p*-value<0.05 was considered to indicate statistical significance.

## RESULTS

### Patient characteristics

**Figure 1:**
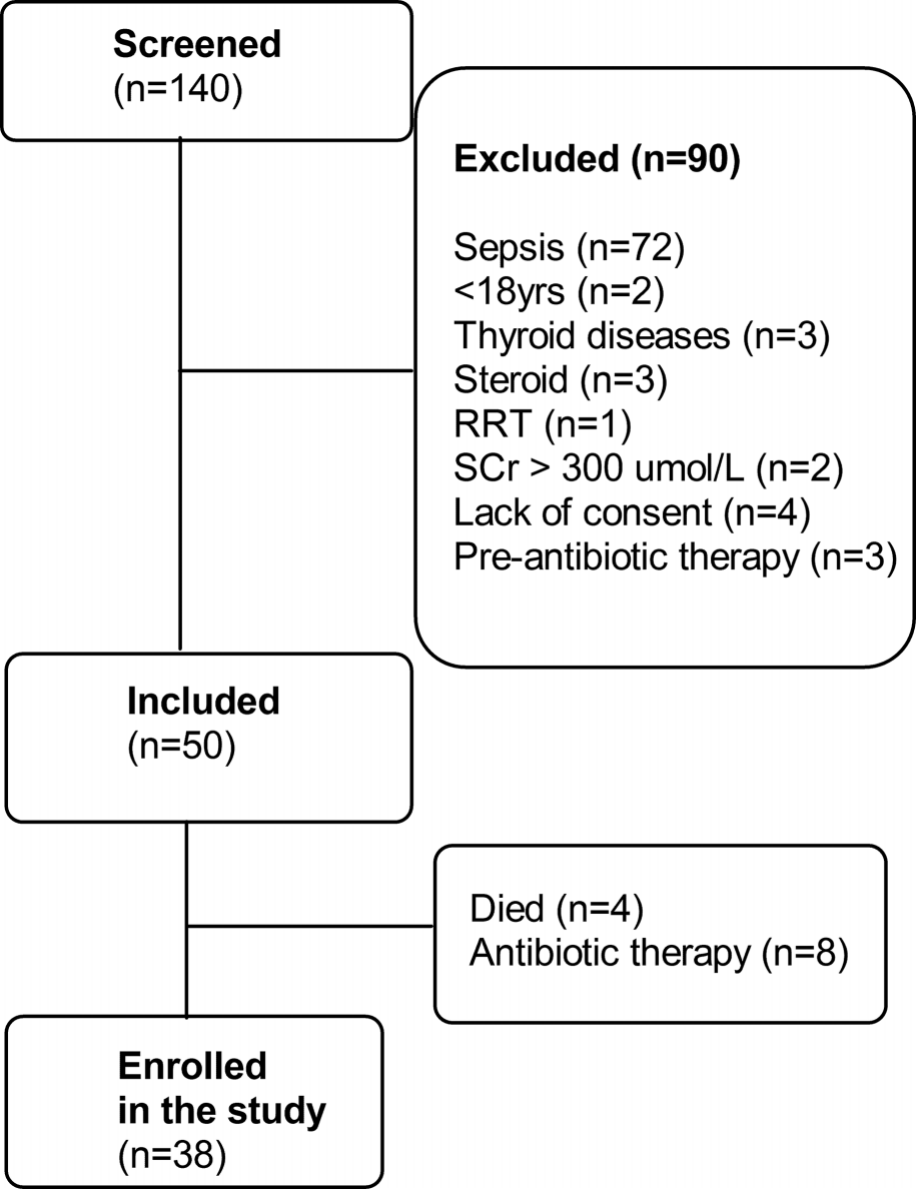
Flow chart depicting the selection process. RRT: renal replacement therapy; SCr: serum creatinine.

A total of 140 prospective patients requiring a tracheotomy were screened. Of these, 72 subjects with sepsis were excluded according to the sepsis diagnosis criteria and 18 subjects were excluded according to the exclusion criteria. Of the remaining 50 patients, 12 were excluded because 4 subjects died (3 in the PDT group and 1 in the ST group) and 8 subjects received antibiotic therapy (3 in the PDT group and 5 in the ST group) during the observation period. Finally, 38 subjects were eligible for enrollment in the study (Figure 1). The mean age of the study population was 51±16.4 years old, and 20 patients (52.6%) were male.

Among the total included patients, 19 underwent a tracheotomy with the PDT procedure, and the other 19 patients received the ST procedure. There was no significant difference in age, gender, white blood cell (WBC) count, APACHE II score, etiological factors, or underlying diseases (*p*>0.05 for all) (Table 1). The reasons for tracheotomy are shown in an Table 2.No patients experienced complications.

### Dynamic changes in PCT levels after tracheotomy

**Figure 2:**
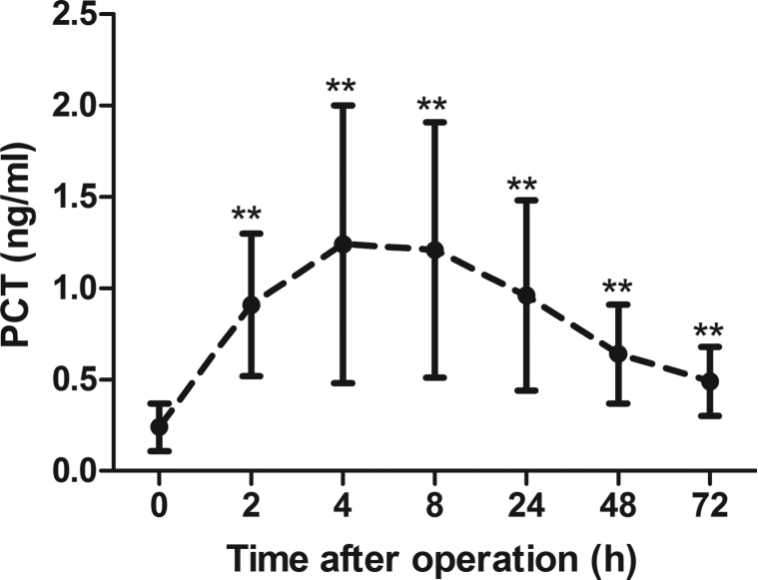
Dynamic changes in PCT levels post-tracheotomy in all patients. Data are presented as the mean±SD. The PCT levels increased immediately after tracheotomy and gradually decreased thereafter. PCT: procalcitonin. **p<0.001 *vs.* T0, obtained using an ANOVA.

To assess the impact of tracheotomy on PCT concentrations in patients without sepsis, the dynamic changes in PCT levels over the observation period were analyzed (Figure 2). The results showed that the PCT levels were significantly higher at each time point after the procedure compared to baseline (*p*<0.001 for all), which was reflected by a linear curve. The curve showed that the levels of PCT increased dramatically after 2 h, from 0.24±0.13 to 0.91±0.39 ng/mL, with a 4-fold increase above baseline (*p*<0.001). The PCT levels reached a peak 4 h later (1.24±0.76 ng/mL), with a 5-fold increase above baseline, which was maintained for approximately 4 h. Then, the levels gradually returned to 2-fold higher than the basal level within 72 h (0.49±0.19 versus 0.24±0.13 ng/mL, *p*<0.001).

### Relationship between peak PCT level and duration of operation

**Figure 3:**
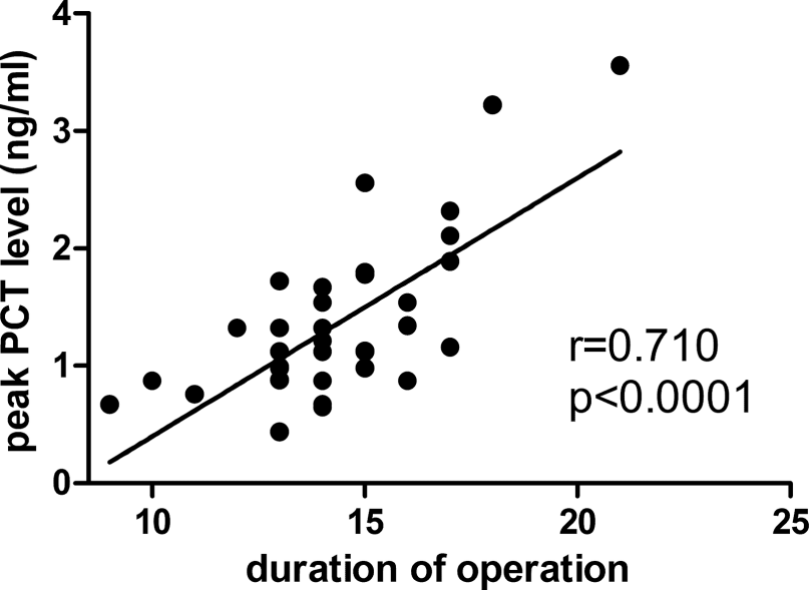
Relationship between peak level of PCT and duration of operation. Based on the Pearson correlation. PCT: procalcitonin.

The PDT group showed a longer operative duration (15.7±1.9 versus 13.2±1.6 min, *p<*0.001). Pearson correlated analysis showed that the peak PCT level did not correlate with the WBC count or APACHE II score (*p=*0.479 and 0.508, respectively), whereas this level had a significant positive correlation with the duration of the tracheotomy in non-septic patients (r=0.710, *p<*0.001) (Figure 3).

### Relationship between PCT concentrations and operative procedure

**Figure 4:**
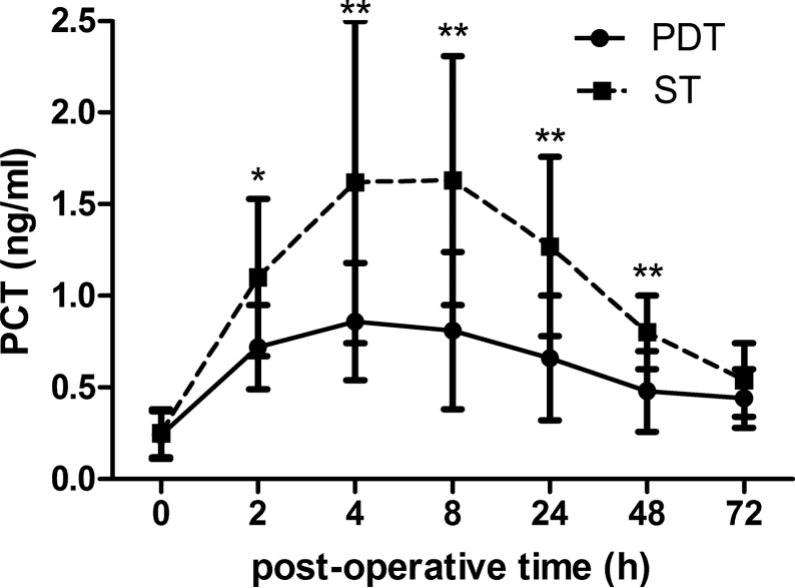
Dynamic changes in PCT level after different operative procedures. Data are presented as the mean±SD. There were significantly higher PCT levels from 2 h to 48 h postoperative in the PDT group compared to the ST group. PCT: procalcitonin; PDT: percutaneous dilatational tracheotomy; ST: surgical tracheotomy. Compared with ST group, * *p*<0.01, ** *p*<0.001.

**Figure 5:**
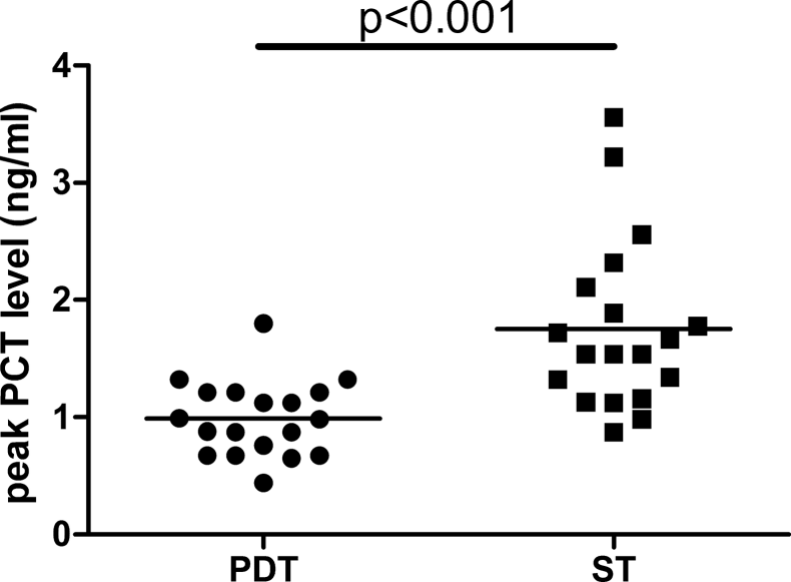
The peak PCT concentrations in the PDT and ST groups. Data are presented as the mean±SD. PCT: procalcitonin; PDT: percutaneous dilatational tracheotomy; ST: surgical tracheotomy. Analysis of the student's *t* test, *p<*0.001.

To evaluate the impact of the operative procedure on PCT concentrations, we analyzed the difference in time course and peak level of PCT between the two groups. In the PDT group, significant increases in the PCT level were observed compared to the ST group from 2 to 48 h after tracheotomy (*p<*0.001 for all) (Figure 4). The results of the repeated ANOVA measurement showed that the time courses in the ST group were significantly different than those in the PDT group, in which there was an interaction effect between time and the operative procedure (F=11.906, *p<*0.001). The patients who underwent tracheotomy via the ST procedure showed a significantly higher peak PCT concentration than those who received the PDT procedure (0.98±0.32 versus 1.77±0.72 ng/ml, *p<*0.001) (Figure 5). Spearman correlation analysis showed that the peak level of PCT was closely associated with the tracheotomy procedure (rho=0.670, *p<*0.001).

## DISCUSSION

The present study found that tracheotomy induced a rapid increase in the serum PCT concentration in patients without sepsis and that the peak level of PCT showed a significant positive correlation with the duration of the operation. Moreover, our results demonstrated that the procedure used to perform the tracheotomy affected the PCT concentration.

In critically ill patients, PCT has been considered a reliable biomarker for diagnosing sepsis and evaluating the severity of disease 1,23,24. In our study, the PCT levels increased immediately after the tracheotomy and gradually decreased to near the basal level. This postoperative dynamic change in PCT levels is supported by other surgical studies 3,. Kaido et al. 27 and Perrakis et al. 28 reported that serum PCT levels increased remarkably after liver transplantation, with a peak at postoperative day 2 or 3 and a gradual decrease thereafter. A recent cohort study by Silvestre et al. 25 reported that the PCT levels increased immediately and peaked at 24 to 48 h after colorectal surgery and gradually decreased thereafter and that the PCT time course was virtually identical in postoperative patients with or without infection. Meyer et al. 2 reported that an increase in PCT levels did not help to predict surgical infections in critically ill surgical patients. Together, these results show that the postoperative PCT levels increase not as a response to infection but rather for other reasons, which are described as follows: (1) Due to the anatomy of the operating site in a tracheotomy, the procedure is carried out adjacent to the thyroid gland, and the relationship between the circulatory, metabolic, nervous and endocrine systems is regulated and affected by the thyroid gland. Thus, an operative procedure to the neck area causes surgical stress, with a corresponding increase in thyroid hormones or even a thyroid crisis. This hypothesis is supported by previous studies 19, 20,29. (2) In both previous studies and the current study, operative stress was found to affect short-term PCT release. In the present study, the operative duration and procedure showed clear impacts on the peak level of PCT, although the precise correlation between surgical trauma and PCT release is not fully understood.

Meisner et al. 26 suggested that peak PCT levels increased within 24 h postoperative and returned to normal levels within the first week after surgery, depending on the degree of PCT elevation during the procedure and the type of the surgical procedure used. This finding may explain why the operative duration and procedure showed evident impacts on the peak levels of PCT in this study. Moreover, a tracheotomy is a brief surgery, which may help to explain why in our study, the PCT levels peaked earlier and the peak PCT levels were lower than those reported in previous studies. Compared to the ST procedure, the PDT procedure has advantages related to the duration of the operation and the degree of trauma. Moreover, interference with the thyroid gland may be more likely during the ST than the PDT procedure. Esen et al. 20 reported that the thyroid hormones in patients who underwent the ST procedure showed a significantly greater increase than in patients who underwent the PDT procedure. On the other hand, in the present study, the duration of operation in the PDT group was shorter compared to the ST group (Table 1). This result may explain why patients who underwent the ST procedure had a higher peak PCT level than patients who received the PDT procedure.

To the best of our knowledge, this is the first study to investigate the impact of tracheotomy on the PCT concentration. However, the nonspecific elevated PCT level after surgery in patients without sepsis can be a challenge in clinical decision-making. Thus, physicians need to consider sepsis in the diagnosis when determining whether to initiate antibiotic therapy. Given the impact of surgical stress, the PDT procedure should be preferred to the ST procedure.

Certain limitations of this study should be considered. First, this was a single-center study, and the number of patients included was relatively small. Larger prospective studies are thus required to confirm these findings. Second, the present study observed the dynamic change trend in PCT concentrations over 72 h, and whether the short-term increase in post-operative PCT levels affects the incidence of follow-up sepsis remains unclear. Third, the development of sepsis is a multifactorial process that is difficult to detect with any single biomarker. In our study, we only assessed the impact of tracheotomy on PCT concentrations, excluding classic inflammatory biomarkers such as WBCs and C-reactive protein (CRP). Thus, it is difficult to explain the mechanisms by which a tracheotomy affects short-term serum PCT increases based on a single factor.

In summary, our study demonstrated that tracheotomy induces a rapid increase in serum PCT in patients without sepsis, and the operative duration and procedure have clear impacts on the peak levels of PCT. Thus, the nonspecific increase in PCT level after a tracheotomy needs to be considered when it is used to aid physicians in the diagnosis of sepsis.

## Figures and Tables

**Table 1:** Baseline characteristics of patients without sepsis.

Characteristics	All patients(n=38)	PDT group(n=19)	ST group(n=19)	*p*-value^a^
**Age (years)**	51±16.4	49.3±16.7	52.9±16.4	0.508
**Gender (M/F)**	20/18	8/11	12/7	0.194
**WBC (×109/L)**	9.03±3.66	9.00±3.29	9.04±4.08	0.972
**APACHE II score**	17.1±2.0	16.8±1.93	17.5±1.98	0.288
**Etiological factors (n, %)**				0.893
Cerebrovascular accident	13(34.2)	6(31.5)	7(36.8)	
ACS	9(23.7)	5(26.3)	4(21.1)	
Trauma	7(18.4)	3(15.8)	4(21.1)	
Post-cardiopulmonary resuscitation	4(10.5)	3(15.8)	1(5.3)	
Tetanus	3(7.9)	1(5.3)	2(10.5)	
Guillain-Barre syndrome	2(5.3)	1(5.3)	1(5.3)	
**Underlying diseases (n, %)**				0.949
Hypertension	8(21.1)	5(26.3)	3(15.8)	
Diabetes	6(15.8)	3(15.8)	3(15.8)	
Coronary heart disease	6(15.8)	4(21.1)	2(10.5)	
COPD	5(13.2)	3(15.8)	2(10.5)	
Nervous system disease	4(10.5)	3(15.8)	1(5.3)	

Quantitative data with a normal distribution are presented as the mean±SD. Qualitative data are presented as n (%). The ^a^*P* values were derived from the Chi-square test for categorical data and the student's *t*-test for independent samples. ACS: Acute coronary syndrome; COPD: obstructive pulmonary disease.

**Table 2:** Reasons for tracheostomy among the study participants.

Patient No.	Diagnosis	Reason
1	cerebral hemorrhage	coma, LTMV
2	high paraplegia	LTMV
3	high paraplegia	LTMV
4	HIE	coma, LTMV
5	cerebral hemorrhage	coma, cerebral hernia
6	Guillain-Barre	LTMV
7	cerebral infarction	coma, LTMV
8	severe thoracic wound	sedation, LTMV
9	cerebral hemorrhage	coma, dyspnea
10	pulmonary heart disease	LTMV
11	refractory heart failure	LTMV
12	refractory heart failure	LTMV
13	tetanus	NMBAs, LTMV
14	cerebral infarction	LTMV
15	cardiogenic shock	LTMV
16	cerebral hemorrhage	coma, dyspnea
17	AMI	LTMV
18	high paraplegia	LTMV
19	HIE	coma, LTMV
20	cerebral infarction	LTMV
21	tetanus	NMBAs, LTMV
22	cerebral hemorrhage	coma, dyspnea
23	cardiogenic shock	LTMV
24	pulmonary heart disease	LTMV
25	HIE	coma, LTMV
26	HIE	sedation, LTMV
27	cerebral hemorrhage	glossocoma, coma, LTMV
28	Guillain-Barre	LTMV
29	severe thoracic wound	sedation, LTMV
30	refractory heart failure	LTMV
31	cerebral infarction	glossocoma, LTMV
32	severe thoracic wound	sedation, LTMV
33	traumatic brain injury	sedation, LTMV
34	cerebral hemorrhage	coma, dyspnea
35	high paraplegia	LTMV
36	respiratory failure	LTMV
37	tetanus	NMBAs, LTMV
38	cerebral infarction	LTMV

AMI: acute myocardial infarction; HIE: hypoxic-ischemic encephalopathy; LTMV: long-term mechanical ventilation; NMBAs: neuromuscular blocking agents
